# Left ventricular outflow tract obstruction in transthyretin amyloid cardiomyopathy: a case report on diagnostic and treatment challenges and role of alcohol septal ablation

**DOI:** 10.1093/ehjcr/ytaf233

**Published:** 2025-05-12

**Authors:** Ali Hussein Jaber Mejren, Sie Kronborg Fensman, Steen Hvitfeldt Poulsen

**Affiliations:** Department of Cardiology, Aarhus University Hospital, Palle Juul-Jensens Boulevard 99, Aarhus N 8200, Denmark; Department of Cardiology, Aarhus University Hospital, Palle Juul-Jensens Boulevard 99, Aarhus N 8200, Denmark; Department of Cardiology, Aarhus University Hospital, Palle Juul-Jensens Boulevard 99, Aarhus N 8200, Denmark

**Keywords:** Transthyretin amyloid cardiomyopathy, Left ventricular outflow tract obstruction, Alcohol septal ablation, Case report

## Abstract

**Background:**

Transthyretin amyloid cardiomyopathy (ATTR-CM) is a restrictive cardiomyopathy caused by amyloid deposition in the myocardium. Its phenotypical overlap with hypertrophic cardiomyopathy, particularly in cases involving left ventricular outflow tract obstruction (LVOTO), challenges accurate diagnosis. Medical management of LVOTO in ATTR-CM is challenged by the opposing effects of beta-blockers and diuretics.

**Case summary:**

A 79-year-old male with left ventricular hypertrophy and LVOTO presented with worsening dyspnoea. Full diagnostic work-up confirmed wild-type ATTR-CM. A conservative medical approach with diuretics and beta-blockers proved challenging. Alcohol septal ablation was successfully performed without major complication, resolving the LVOTO and improving symptoms.

**Discussion:**

The diagnosis and management of ATTR-CM with LVOTO are complex. A thorough diagnostic approach is needed to avoid mismanagement. Diuretics and beta-blockers must be carefully balanced to achieve optimal clinical results. Alcohol septal ablation may be considered in patients with persistent symptoms and high LVOT gradients despite optimal medical therapy.

**Learning points**

- Differentiating transthyretin amyloid cardiomyopathy (ATTR-CM) from hypertrophic cardiomyopathy can be challenging, especially in the presence of left ventricular outflow tract obstruction (LVOTO). A multi-modality imaging approach may be necessary.
- Managing LVOTO in ATTR-CM patients presents unique challenges as the recommended treatments—beta-blockers for LVOTO and diuretics for ATTR-CM—can exert opposing effects. A carefully balanced approach is necessary to achieve optimal treatment outcomes.
- Alcohol septal ablation may be considered on a case-by-case basis in ATTR-CM patients with LVOTO and persistent symptoms despite optimal medical therapy.

## Introduction

Transthyretin amyloid cardiomyopathy (ATTR-CM) is an infiltrative restrictive cardiomyopathy caused by amyloid fibril deposition in the myocardium. This leads to thickened left ventricular (LV) walls, reduced LV cavity size, and systolic and diastolic dysfunction. Transthyretin amyloid cardiomyopathy has been detected in ∼17% of elderly patients with heart failure (HF) and septal hypertrophy.^1^

The echocardiographic phenotype in ATTR-CM often mimics hypertrophic cardiomyopathy (HCM).^2^ Myocardial thickening in ATTR-CM is often most prominent in the basal septum, which can cause dynamic LV outflow tract obstruction (LVOTO) in a small subset of cardiac amyloidosis patients.^3,4^ This phenotypical overlap can challenge the differentiation between ATTR-CM with LVOTO and obstructive HCM (HOCM).

Treatment of ATTR-CM and HOCM differs due to the distinct underlying pathophysiological mechanisms. In co-existing ATTR-CM and LVOTO cases, management requires a carefully balanced approach using diuretics and beta-blockers. We present a case of LVOTO in a patient with presumed wild-type ATTR-CM, highlighting diagnostic and therapeutic challenges and evaluating the safety and efficacy of transcatheter alcohol septal ablation (ASA).

## Summary figure

**Figure:**
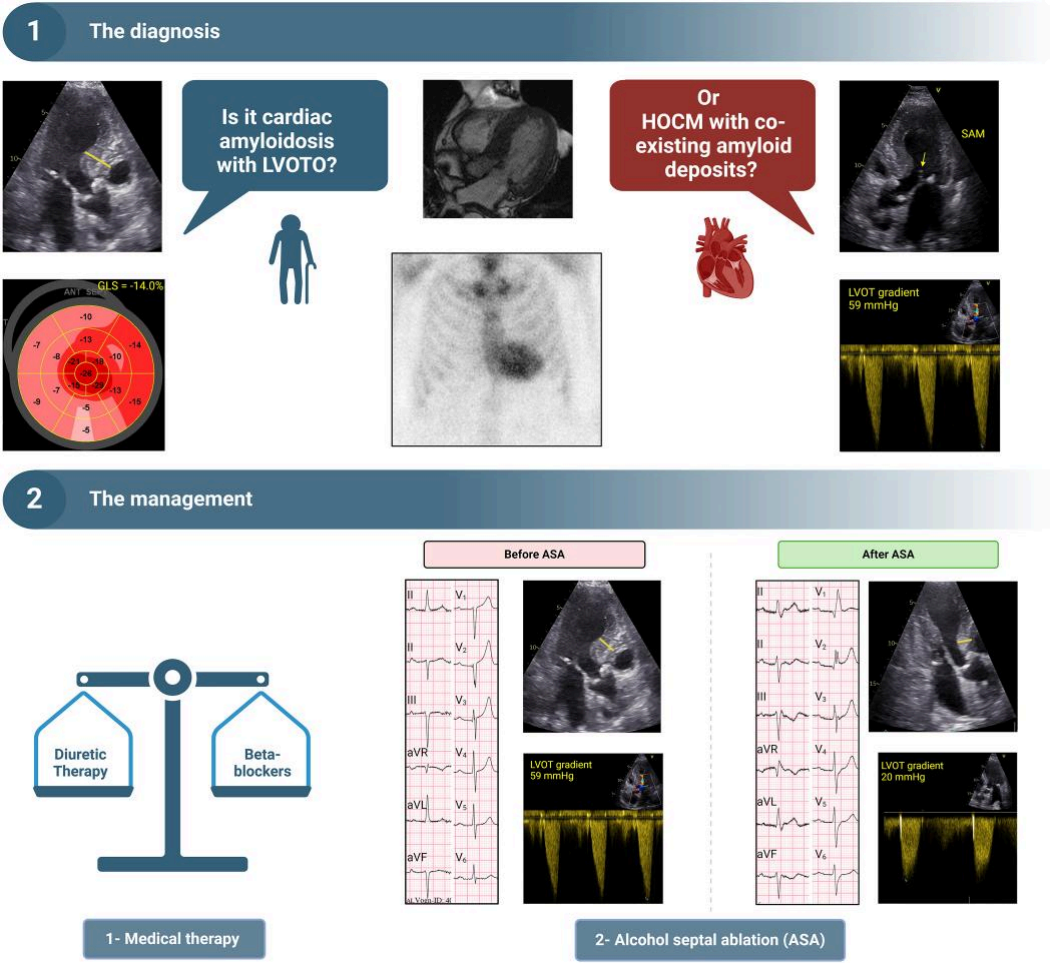


## Case presentation

A 79-year-old male patient with hypertension and well-controlled moderate asthma presented with progressive dyspnoea, consistent with New York Heart Association (NYHA) Class II, and increasing leg oedema over 6 months. Baseline treatment included losartan/hydrochlorothiazide 50 + 12.5 mg/day, beta-2 agonist, and steroid inhalations.

Initial transthoracic echocardiography demonstrated significant LV hypertrophy measured 20 mm in the septum, preserved LV ejection fraction (LVEF) of 60%, systolic anterior motion of the mitral valve (SAM), and mild mitral regurgitation. Doppler flow over the LV outflow tract (LVOT) showed classic dagger-shaped curves with a maximum resting gradient of 36 mmHg, suggesting HOCM. Losartan/hydrochlorothiazide was discontinued, and the patient was referred to our tertiary cardiac centre.

**Figure 1:**
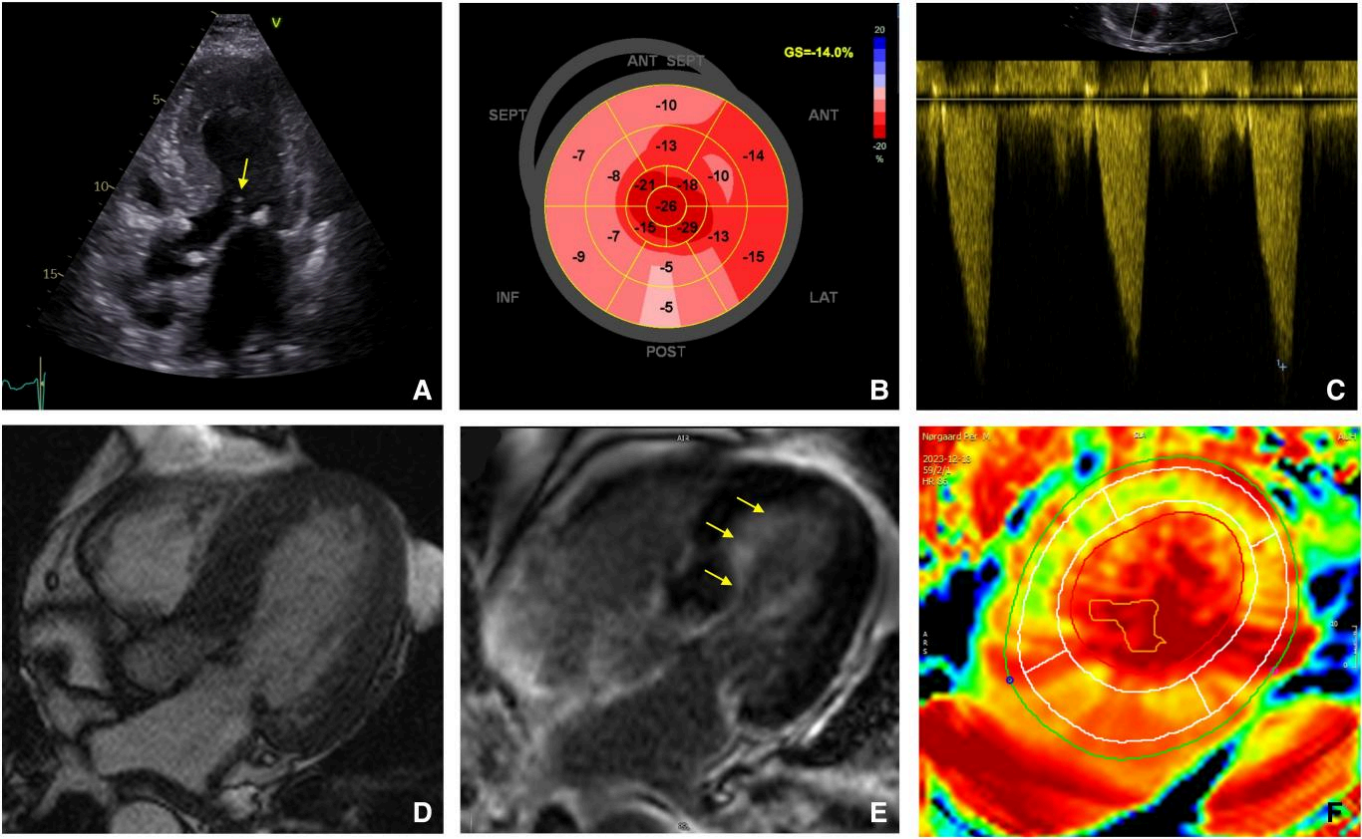
Imaging. (*A–C*) Echocardiography images: (*A*) apical five-chamber view displaying systolic anterior motion of the mitral valve (arrow). (*B*) Bull’s-eye map of the left ventricular global longitudinal strain pattern with relative apical sparing. (*C*) Continuous wave Doppler showing typical dagger-shaped signal of left ventricular outflow tract gradient. (*D–F*) Cardiac magnetic resonance images. (*D*) Cine Cardiac magnetic resonance image showing asymmetrical left ventricular hypertrophy. (*E*) Inversion recovery sequence showing late gadolinium enhancement with subendocardial late gadolinium enhancement pattern (yellow arrows). (*F*) Extracellular volume map.

At our centre, transthoracic and transoesophageal echocardiography showed LVOT flow acceleration by colour Doppler, with an LVOT gradient of 15 mmHg at rest and 31 mmHg during Valsalva. No SAM was detected. Global longitudinal strain (GLS) was −16.6% with a relative apical sparing (RELAPS) pattern (*Figure 1A–C*).

Cardiac magnetic resonance (CMR) revealed severe LV hypertrophy, preserved LVEF, and subendocardial late gadolinium enhancement that did not follow an ischaemic pattern (*Figure 1D–F*). Detailed CMR measurements are shown in *Table 1*.

**Table 1:** Cardiac magnetic resonance imaging

Parameter	Result	Normal range^a^
EDV (mL)	84.4	95–215
EDV index (mL/m^2^)	42.3	50–108
ESV (mL)	20.7	25–85
ESV index (mL/m^2^)	10.3	11–47
LVEF (%)	75.5	49–79
LV mass (g)	207.3	66–176
LV mass index (g/m^2^)	103.8	39–85
Maximal LV wall thickness (mm)	24.2	≤11
Native T1, total (ms)	1091 ± 61	905–1073
ECV (%)		
- Total	37 ± 8	17–29
- Basal section	31.7	
- Middle section	36.0	
- Apical section	42.6	

ECV, extracellular volume; EDV, end-diastolic volume; EF, ejection fraction; ESV, end-systolic volume; LV, left ventricle.

^a^Reference for normal range values.^5^

Transthyretin amyloid cardiomyopathy was suspected due to several red flags, including advanced age, male sex, and slightly reduced GLS with RELAPS. Classic low-voltage electrocardiogram (ECG) was absent; however, discordance between normal QRS voltage and severe LV hypertrophy raised suspicion. Other common red flags, such as carpal tunnel syndrome, spinal stenosis, and peripheral neuropathy, were absent. Wild-type ATTR-CM diagnosis was confirmed by high myocardial uptake on ^99m^Tc- 3,3-diphosphono-1,2-propanodicarboxylic acid (DPD) scintigraphy (Perugini Grade 3), normal plasma free-light chain quantification, and absence of plasma and urine monoclonal protein. Endomyocardial biopsies showed Congo-red positive amyloid deposition, confirmed by mass spectrometry as ATTR. There were no signs of myocardial disarray. Genetic testing was negative for mutations associated with hereditary ATTR-CM and HCM. Oral furosemide 40 mg/day was initiated due to leg oedema.

One month later, there was no clinical improvement. Echocardiography showed a peak resting LVOT gradient of 59 mmHg, and SAM reoccurred with mild mitral regurgitation. Global longitudinal strain decreased further to −14%. Furosemide was reduced to 20 mg/day, and metoprolol 50 mg/day was initiated.

While attempting to balance diuretics and beta-blockers, the patient’s symptoms remained poorly controlled and progressively worsened to NYHA Class III. Left ventricular outflow tract gradients increased to 85 mmHg at rest and 105 mmHg during Valsalva. In response, metoprolol was increased to 100 mg/day. Although LVOT gradients decreased, the patient’s condition deteriorated, leading to hospitalization a few days later due to decompensated HF. Intravenous diuretics and pleural drainage resulted in a significant clinical improvement (NYHA Class II) at discharge.

Following discharge, the persistence of LVOTO, combined with the continued need for diuretic therapy, prompted the decision to perform ASA.

Alcohol septal ablation was performed without complications, resolving LVOTO and reducing proximal septal wall thickness. However, the patient developed a post-ASA right bundle branch block in addition to a pre-existing left anterior fascicular block and first-degree atrioventricular (AV) block. No advanced AV block was detected neither during monitoring at hospital admission nor during post-ASA Holter.

**Figure 2:**
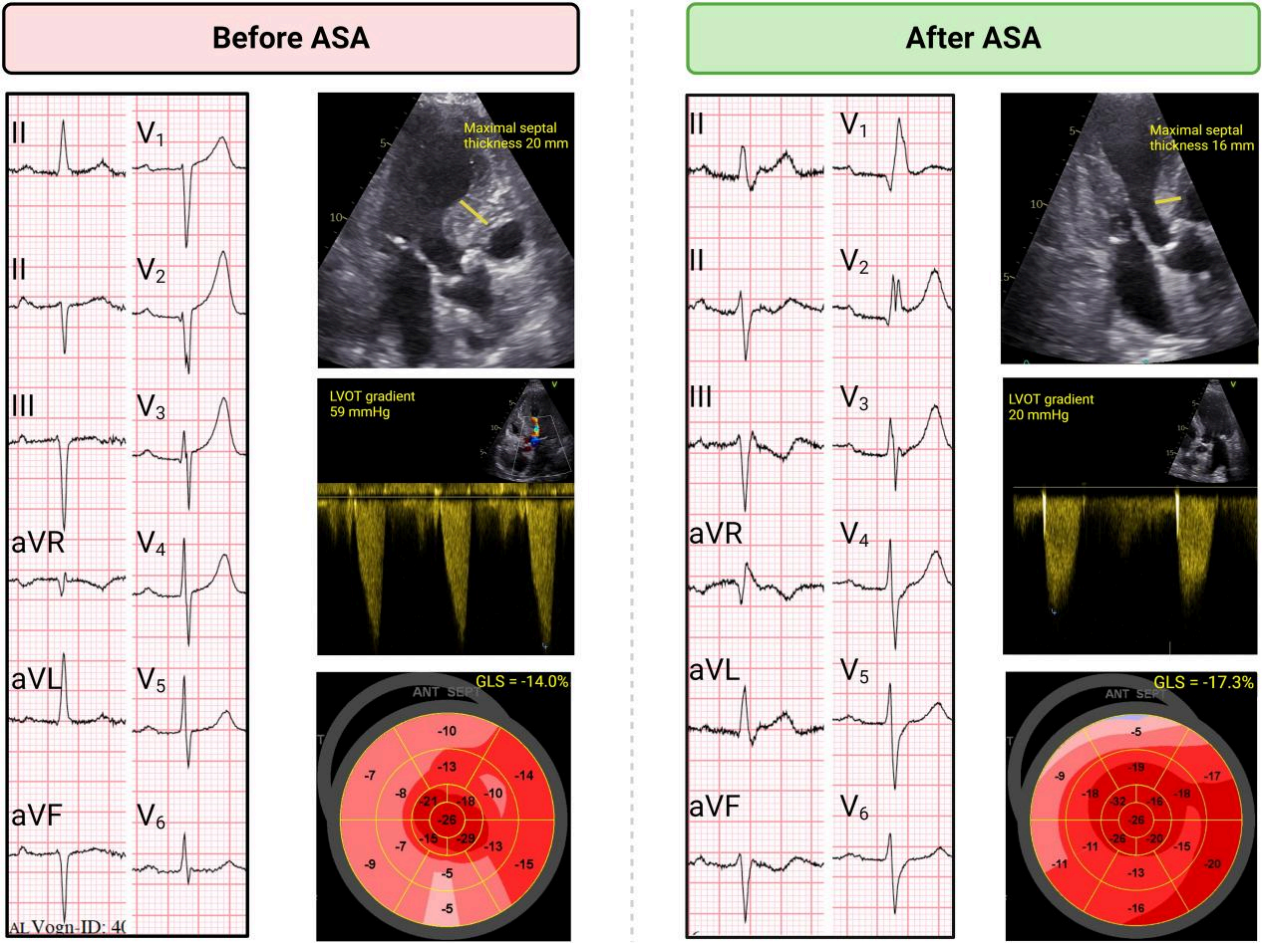
Before and after alcohol septal ablation. ASA, alcohol septal ablation; GLS, global longitudinal strain; LVOT, left ventricular outflow tract.

At a 5-month post-ASA clinical follow-up, the patient reported significant improvement, with almost no functional symptoms, thus classified as NYHA Class I. Echocardiography showed no signs of increased LVOT gradients or SAM, neither at rest nor during Valsalva, preserved LVEF, and significant improvement in GLS (−17.3) (*Figure 2*).

## Discussion

This case illustrates the complexities of diagnosing and managing ATTR-CM patients when complicated by co-existing LVOTO.

The first challenge was to establish the correct diagnosis. The initial echocardiography suggested HOCM, but a positive DPD scintigraphy (Perugini Grade 3) contradicted this perception. A key dilemma was whether this was HOCM with co-existing amyloid deposits or cardiac amyloidosis with concurrent LVOTO. Helder *et al*.^6^ identified amyloid deposits in septal myomectomy specimens of 0.9% of patients undergoing septal myomectomies for HOCM. However, the authors concluded that these mild deposits were insufficient to explain LVOTO, thus reinforcing the primary diagnosis of HOCM. In contrast, our patient had biopsy-confirmed ATTR depositions and Perugini Grade 3 DPD scintigraphy, indicative of a high amyloid burden.^7^ Coupled with reduced GLS and RELAPS, negative genetic testing for HCM, patient's sex, and advanced age, these findings strongly favour a diagnosis of wild-type ATTR-CM.^2,8^

Notably, CMR revealed the highest extracellular volume values in the apex. This may contradict the theory of a base-to-apex amyloid gradient as the cause of RELAPS.^9^ However, De Gaspari *et al*.^10^ suggested that RELAPS pattern may not reflect a base-to-apex amyloid gradient but rather a more complex epiphenomenon.

The next challenge was developing an appropriate treatment plan. Transthyretin amyloid cardiomyopathy is characterized by elevated filling pressures at rest and especially during exercise, which usually leads to clinical signs of fluid overload requiring diuretic therapy.^11^ In contrast, diuretics are relatively contraindicated in HOCM as they may exacerbate LVOTO due to small LV cavity and hypercontractile myocardium. Beta-blockers are first-line therapy for HOCM due to their ability to decrease contractility and heart rate, allowing for better diastolic filling, and minimizing LVOTO.^12^ However, beta-blockers are generally avoided in ATTR-CM as the condition is associated with low stroke volume, necessitating a higher heart rate to maintain adequate cardiac output.^13^

**Figure 3:**
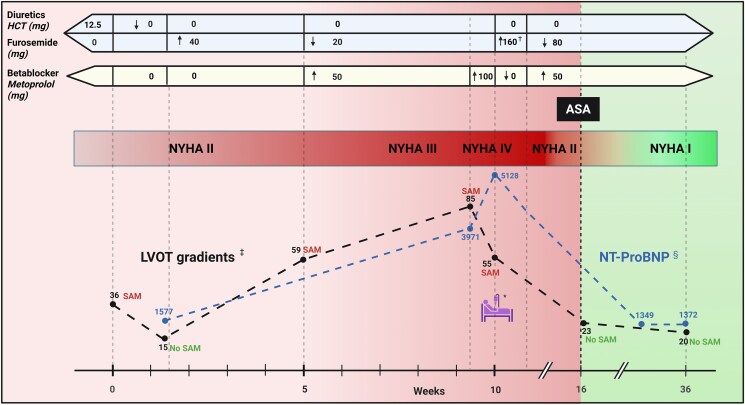
Timeline. *Admission to the ward because of decompensation. †Intravenous diuretics. ‡Resting left ventricular outflow tract gradients (mmHg). §Normal range for N-terminal pro-B-type natriuretic peptide <300 ng/L. ASA, alcohol septal ablation; HCT, hydrochlorothiazide; LVOT, left ventricular outflow tract; NT-proBNP, N-terminal pro-B-type natriuretic peptide; NYHA, New York Heart Association; SAM, systolic anterior motion of the mitral valve.

This opposing effect of treatment strategies was demonstrated in our patient. Left ventricular outflow tract obstruction was highly sensitive to changes in preload. Flow acceleration and SAM were detected whenever the patient was on diuretic therapy and resolved or minimized when diuretics were discontinued. However, reducing diuretics likely worsened hypervolaemia. Although metoprolol reduced LVOT gradients, it may have further contributed to clinical deterioration due to its negative inotropic and chronotropic effects (*Figure 3*).

Given these considerations, ASA was deemed the best treatment option. Data on efficacy and safety of ASA in this subset of patients are limited. However, this case demonstrated the long-term efficacy and safety of ASA in managing this complex case as it ensured complete resolution of LVOTO without major complications.

Alcohol septal ablation has been reported in a few cases of LVOTO in cardiac light chain amyloidosis, with satisfactory clinical results and without safety concerns.^14,15^ To our knowledge, this is the first case report describing ASA in ATTR-CM patients. This emphasizes the need for further research to explore and validate treatment strategies for managing LVOTO in ATTR-CM.

## Data Availability

All available data underlying this article are presented within the manuscript.

## Lead author biography

Sie K. Fensman is a medical doctor and PhD student at the Department of Cardiology, Aarhus University Hospital. Her research focuses on wild-type transthyretin amyloid cardiomyopathy (ATTRwt-CM), an under-recognized condition caused by amyloid protein deposits in the heart. She has contributed to several studies on cardiac amyloidosis, exercise haemodynamics, and device-related outcomes. Through her observational research, Dr Fensman aims to enhance the understanding, diagnosis, and management of ATTRwt-CM. Her work bridges clinical practice and academic research, advancing both patient care and scientific knowledge of this complex disease.
